# Schuylkill Valley

**Published:** 1897-07

**Authors:** Otto Noack

**Affiliations:** Corresponding Secretary


### SCHUYLKILL VALLEY VETERINARY MEDICAL ASSOCIATION.

The annual meeting was held on Wednesday, June, 16, 1897, at the Hotel Woll, Pottsville, Pa. The meeting was called to order by Dr. Sallade at 11 A.M., who acted as temporary chairman, President Burkholder being in Chicago filling the duties of meat-inspector in the employ of the United States Government. Dr. Sallade, in order to make the new members acquainted with the Constitution and By-laws, read them first. To roll-call the following members responded: Bieber, McCarthy, Noack, Sallade, Schneider, Yingst. The minutes of previous meeting were read and approved. Under admission of new members, the names of Drs. Longacre, of Shenandoah, and Newhard, of Ashland, were presented, by the Secretary and their applications handed to the Trustees for examination. After recess of a few minutes, these gentlemen were reported favorably, and, after report, duly elected to membership.

Dr. Newhard was also present, and was introduced to the members. Under deferred business, the questions what should be done with delinquents for non-payment of dues was disposed of in this way, that Hagenbush be expelled, and the names of Drs. Fridirici and Snyder were dropped conditionally from the list of membership. After this the Treasurer read his report, showing that the Society was indebted to members to the amount of $25.75, and due to the Society for dues and initiation fees $33.89.

The report was examined by the Trustees and approved. Under head of new business, communications were read and received, followed by the nomination and election of new officers: President, Dr. Otto Noack, Reading; Vice-President, Dr. Schneider, Ashland; Recording-Secretary, Dr. Bieber, Kutztown; Corresponding-Secretary, Dr. Sallade, Pottsville; Treasurer, Dr. McCarthy, Pottsville; Trustees, Drs. Brackbill, Reading; Yingst, Shenandoah; Newhard, Ashland. Dr. Yingst read his paper, "Pathological Shoeing," which was discussed and followed by the reports of cases, "Tetanus in a Cat," and "Bursitis Intertubercularis," by Dr. Noack;

"Tetanus in Mule, and its Treatment with Gelsemium," by Dr. Newhard; "Parturient Apoplexy," by Dr. Schneider.

Papers for next meeting will be read by Drs. Schneider and Newhard. Meeting adjourned at 4.30 P.M., and agreed to meet in Reading in September.

OTTO NOACK,
Corresponding Secretary.

